# Multibeam bathymetry data of discovery gap in the eastern North Atlantic

**DOI:** 10.1016/j.dib.2020.105679

**Published:** 2020-05-13

**Authors:** Ivan Dudkov, Evgenia Dorokhova

**Affiliations:** a Shirshov Institute of Oceanology, Russian Academy of Sciences; b Immanuel Kant Baltic Federal University

**Keywords:** Multibeam swath bathymetry, Digital elevation model, Deep ocean gateway, Geomorphology

## Abstract

We present a multibeam bathymetry dataset of Discovery Gap; a narrow gap in ridge of the Azores-Gibraltar Fracture Zone (eastern North Atlantic). The gap serves as gateway for the exchange of deep water between the Madeira and Iberian abyssal plains. A bathymetric survey was carried out during the 43^rd^ cruise of the research vessel (R/V) *Akademik Nikolaj Strakhov* in October 2019. The data were collected with the acquisition system RESON SeaBat 7150 (12.5 kHz), using the integrated navigation system Applanix POS MV, and processed using QINSy software. The multibeam bathymetry data are presented in tabular format (ASCII-table (*.txt), spreadsheets (*.xlsx)), and as a digital elevation model (DEM) of the bottom relief. The DEM was created using ArcGIS software in ESRI-ASCII-grid (*.asc) and GeoTIFF raster (*.tif) formats with a spatial resolution of 100 m. We provide processed data that can be used directly for further studies. The dataset is available with the article.

Specifications table

**Table:** 

Subject	Geology
Specific subject area	Bathymetry, seafloor geomorphology, oceanography
Type of data	Tabular data, digital elevation model (DEM) of sea bottom relief
How data were acquired	Field survey, shipboard acquisition system. Multibeam echo-sounder RESON Seabat 7150, frequency 12.5 kHz.
Data format	Tabular data: ASCII-table (*.txt), spreadsheets (*.xlsx). Digital elevation model of sea bottom relief: ESRI-ASCII-grid (*.asc), GeoTIFF raster (*.tif).
Parameters for data collection	Vessel speed 6 knots during the multibeam survey. The survey was designed as a series of parallel swath with 30-40% of overlap.
Description of data collection	The raw multibeam data was processed using QINSy software (QPS, Netherlands). Validated multibeam data were converted in the ASCII- table (*.txt) and to spreadsheets (*.xslx). A DEM was created by triangular method and converted into ESRI-ASCII-grid (*.asc) and GeoTIFF raster (*.tif) in QINSy and ArcGIS software.
Data source location	eastern North Atlantic Ocean, Discovery Gap (37^o^ N, 16^o^ W).
Data accessibility	With the article

## Value of the data

• We present new multibeam swath bathymetry data of the abyssal ocean gateway. Ocean gateways play an important role in basin evolution and the exchange of water, sediment, and biota from one basin to another. Despite of their importance, little is known about these bathymetric features. The presented data can significantly contribute to the understanding of the morphology and sedimentation regimes of ocean gateways.
• The DEM reveals a well-detailed topographic image of Discovery gap. It allows to define various morphological features of the gap. The most prominent are deeps, sills, ridges, topographic highs, and flattened areas. Erosive and accumulative features caused by action of bottom currents can also be distinguished.
• The dataset could be used for the geomorphological study of the area, sedimentological studies of abyssal gateways, numerical modeling of deep ocean circulation, and habitat mapping.
• The dataset can be used to improve multibeam survey methods in areas of abyssal ocean gateways.

## 1 Data Description

**Fig 1:**
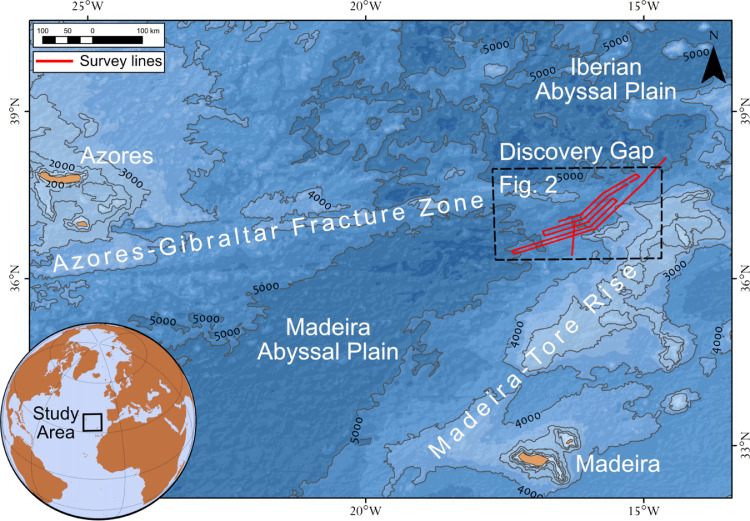
Study area. Bathymetry (depths in m) of the eastern North Atlantic based on the SRTM 15 arc-second global relief [2]. Fig. 2 – location of the Fig. 2.

Discovery Gap is a narrow gap located between the Azores-Gibraltar Fracture Zone and the Madeira-Tore Rise in the eastern North Atlantic (Fig. 1). Discovery Gap lies within the eastern part of the Gloria Fault – one of the major segments of the Azores-Gibraltar Fracture Zone. The area is characterized by high seismic activity [1].

Discovery Gap serves as a gateway between the Madeira and Iberian abyssal plains. Ocean gateways play an important role in water mass exchange and transport of sediment and biota between basins. Previous studies have shown that near-bottom currents are intensified through ocean gateways. This promotes the erosion and accumulation of sediment forming a variety of sea-floor bedforms [3]. A persistent northward flow was registered in Discovery gap [4]. The Gap is seemed to be the terminal point for spreading of Antarctic Bottom Water if it is considered as the water with a potential temperature less than 2°C [5].

The multibeam data were collected during integrated oceanographic studies in Discovery Gap that were held by the Atlantic Branch of the Shirshov Institute of Oceanology, Russian Academy of Sciences (Russia). Measurements were conducted during the 43^rd^ cruise of R/V *Akademik Nikolaj Strakhov* in October 2019.

**Fig. 2:**
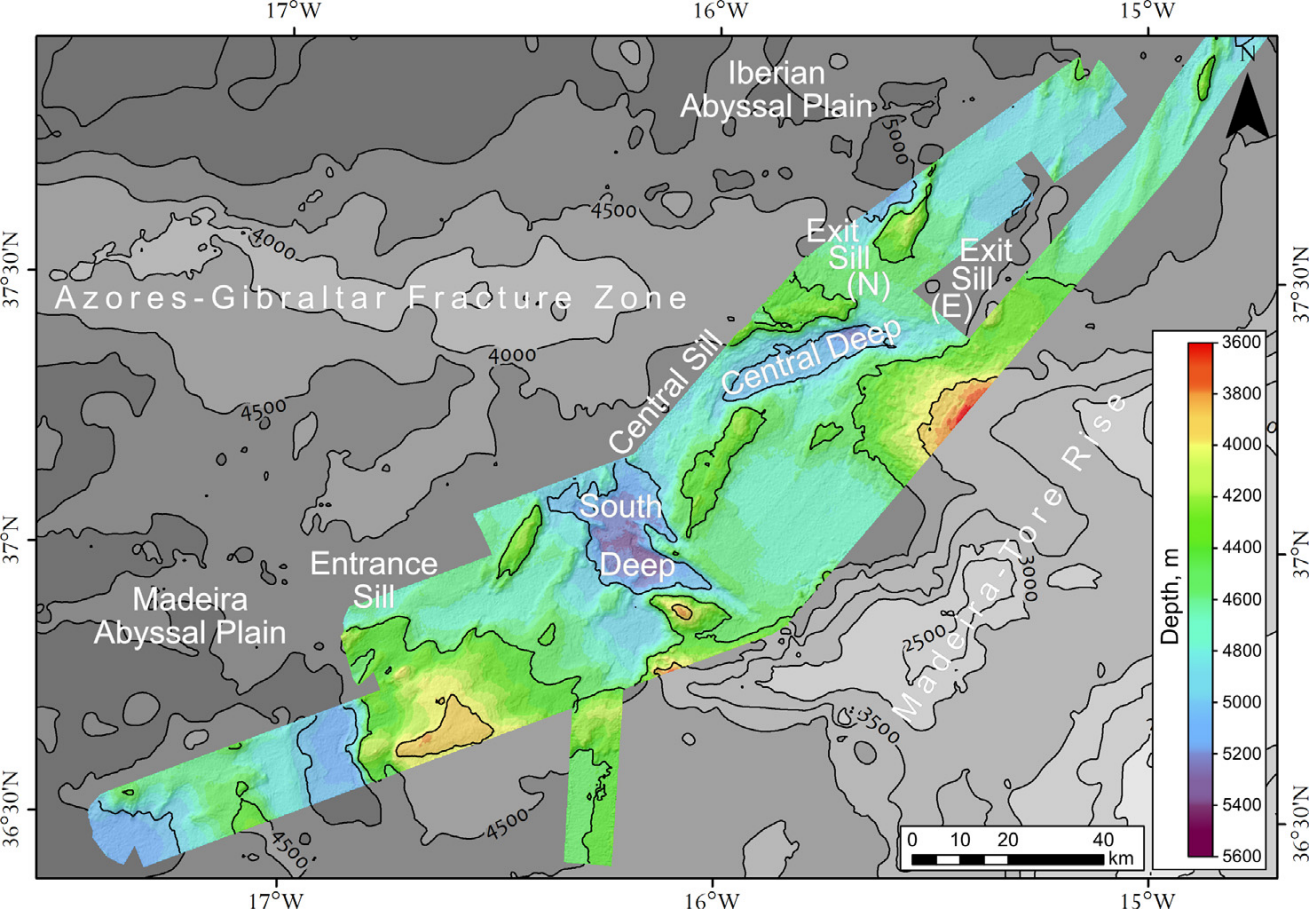
Bathymetry of Discovery Gap (DEM with shaded relief).

According to the collected data, the gap is presented by a variety of pronounced morphological features: deeps, sills, ridges, topographic highs, and flattened areas (Fig. 2). The depths of the sea bottom of the survey area vary between 3800-5400 m with the average depths of 4400-4800 m. The depth of the South Deep of the Discovery Gap is 5000 – 5350 m and the Central Deep is 5000 – 5320 m depth. The Central Sill is 4850 – 4900 m depth and the depth of the flattened area at the eastern part of the Gap is 4600 – 4750 m.

The multibeam bathymetry data are fully processed and presented here in tabular format and as a digital elevation model of the bottom relief. The presented dataset includes:

- An ASCII-table (*.txt) of processed bathymetry data collected by multibeam echosounder Reson SeaBat 7150;
- Spreadsheets (*.xlsx) of processed data, divided into four parts;
- An ESRI-ASCII-grid (*.asc) DEM with a scale 1:100 000, spatial resolution of 100 m in the UTM Zone 28N projection,WGS84;
- A GeoTiff raster (*.tif) of filtered DEM (low pass 3-by-3 filter) (Fig. 2) with a scale of 1:100 000, spatial resolution of 100 m in the UTM Zone 28N projection, WGS84.

The structure of the ASCII-table (*.txt) and MS Excel spreadsheets (*.xslx) is presented in Table 1.

**Table 1:** Description of columns in ASCII-table and MS Excel spreadsheets.

Column Name	Description
System	Echo sounder survey system.
Date	Date of survey, yyyymmdd.
Time	Time of survey (UTC), hhmmss.
Easting	Easting Cartesian coordinate, UTM Zone 28N projection, WGS84.
Northing	Northing Cartesian coordinate, UTM Zone 28N projection, WGS84.
Depth	Corrected and processed depth, m.
Latitude	Geographical latitude, DD;MM.mm, WGS1984.
Longitude	Geographical longitude, DD;MM.mm, WGS1984.

## 2 Experimental Design, Materials, and Methods

The bathymetric survey was carried out by deep sea multibeam echosounder (MBE) system mounted onboard of R/V *Akademik Nikolaj Strakhov*. The acquisition system includes: a multibeam echosounder RESON SeaBat 7150 (12.5 kHz, 256 beams); a navigation system Applanix POS MV with two-antenna GPS receivers (geographical coordinates and true ship heading) and a motion-control sensor; and the data acquisition software, QINSy.

A single swath of MBE data covered a 10 km area, the mean distance between swaths was 80-90 m, the distance between depth points in a single swath was approximately 80 m. The spatial resolution of generated DEM is 100 × 100 m. Any current and wave effects were removed by using a motion-control sensor of Applanix POS MV. The tidal corrections were not applied.

Sound velocity in the water column was calculated from temperature, salinity, and pressure data measured by the CTD-probe SBE19v2+ at the five oceanographic stations in the survey area. Sound velocity profiles were used to correct the multibeam data in the post-processing stage. The multibeam echosounder calibration (Patch Test) was performed.

Data post-processing included the following successive steps: filtration by on-line filters, processing in QINSy, export in ASCII-table, creation of DEM, and conversion to GeoTIFF raster. The depth artifacts were filtered during the survey by the on-line filters in QINSy software (Depth and Range outside interval filters, Single Spike Filter). Data processing in QINSy included: manual filtering of errors, corrections according to the sound velocity in the water column, calculation and application of correction coefficients including the Refract filter for correction of beam refraction, removal of acoustic noise by the off-line filters (Butterworth Smoothing, Adaptive Clip, Spline Surface Despiker) and data filtration in the Qloud software (Qlean++ filter). Then we exported the data of each survey line in XYZ format in ASCII-table. The next step was creation of DEM by triangular method with a spatial resolution of 100 m using QINSy processing manager. Finally, we applied the “Low Filter” function by the module “Spatial Analyst” and converted DEM to GeoTIFF raster using ArcGISsoftware. The resulting bathymetry is presented in UTM 28 zone projection, datum WGS84.

The presented data don't need any pre-processing and may be used directly.

## Declaration of Competing Interest

The authors declare that they have no known competing financial interests or personal relationships which have, or could be perceived to have, influenced the work reported in this article.

## Appendix Supplementary materials

Image, application 1

Image, application 2
